# Rehabilitation-Oriented Serious Game Development and Evaluation Guidelines for Musculoskeletal Disorders

**DOI:** 10.2196/games.7284

**Published:** 2017-07-04

**Authors:** Mohamad Idriss, Halim Tannous, Dan Istrate, Anaick Perrochon, Jean-Yves Salle, Marie-Christine Ho Ba Tho, Tien-Tuan Dao

**Affiliations:** ^1^ Sorbonne University, Université de technologie de Compiègne, CNRS, UMR 7338 Biomechanics and Bioengineering Compiègne France; ^2^ HAVAE EA 6310, Université de Limoges Limoges France; ^3^ Physical and Readaptation Medicine, Hôpital J. Rebeyrol, Limoges University Hospital Limoges France

**Keywords:** rehabilitation exercise, virtual rehabilitation, rehabilitation, user computer interface, musculoskeletal diseases

## Abstract

**Background:**

The progress in information and communication technology (ICT) led to the development of a new rehabilitation technique called “serious game for functional rehabilitation.” Previous works have shown that serious games can be used for general health and specific disease management. However, there is still lack of consensus on development and evaluation guidelines. It is important to note that the game performance depends on the designed scenario.

**Objective:**

The objective of this work was to develop specific game scenarios and evaluate them with a panel of musculoskeletal patients to propose game development and evaluation guidelines.

**Methods:**

A two-stage workflow was proposed using determinant framework. The development guideline includes the selection of three-dimensional (3D) computer graphics technologies and tools, the modeling of physical aspects, the design of rehabilitation scenarios, and the implementation of the proposed scenarios. The evaluation guideline consists of the definition of evaluation metrics, the execution of the evaluation campaign, the analysis of user results and feedbacks, and the improvement of the designed game.

**Results:**

The case study for musculoskeletal disorders on the healthy control and patient groups showed the usefulness of these guidelines and associated games. All participants enjoyed the 2 developed games (football and object manipulation), and found them challenging and amusing. In particular, some healthy subjects increased their score when enhancing the level of difficulty. Furthermore, there were no risks and accidents associated with the execution of these games.

**Conclusions:**

It is expected that with the proven effectiveness of the proposed guidelines and associated games, this new rehabilitation game may be translated into clinical routine practice for the benefit of patients with musculoskeletal disorders.

## Introduction

### Context

Musculoskeletal disorders lead to high medical costs all over the world. These disorders affect the working performance and well-being of the involved people [1,2]. Age-related deficiencies, sport and transport accidents, and genetic conditions are the main sources of these disorders. In the United States, billions of dollars have been spent for treatment and patient management. In Europe, the ageing effect of the population requires significant efforts for medical experts and infrastructures. Research studies have been performed to provide better diagnosis and treatment of these disorders. Among the most common routine practices, functional rehabilitation plays a key role in the recovery of mechanical functions of the human body. This specific treatment helps recover the functionality of the musculoskeletal system by improving the ranges of motion as well as the muscle strengths. Current physical therapy programs are performed by patients and supervised by medical doctors over a long period of time in hospitals or clinics [3,4]. This traditional rehabilitation scheme requires permanent involvement of different medical actors (eg, physiotherapists and medical doctors) during the program, leading to a high cost for medical human resources. Moreover, due to the repetitive nature of the rehabilitation exercises, the motivation of the patient decreases rapidly during the execution of the program. Recently, the progress in information and communication technology (ICT) led to the development of a new rehabilitation scheme called “serious game for functional rehabilitation” [5-7]. In fact, the coupling of the game technologies and functional rehabilitation allows a better interaction between patient and the rehabilitation program [8]. Moreover, the use of serious game scenarios may be a potential solution to improve the patient’s motivation in future rehabilitation sessions.

### State of the Art

Some research studies focused on games that could improve general health for adults and elders. Chen et al (2012) developed a lower limb power rehabilitation system. Each user needs to execute a squat motion, with sufficient power, to correctly build a virtual tower made of blocks [9]. The system was tested with 20 participants, whereas 20 control participants executed normal exercises for 6 weeks. The results showed that the participants using the developed system achieved greater improvements in power and velocity of movement. Sun et al (2013) presented a balance rehabilitation system using Kinect and a force plate [10]. The objective of this game was to fit an avatar in a specific frame indicated on the screen, while standing on the force plate. In total, 23 healthy subjects tested this system, but the results showed that different evaluation methods could lead to different interpretation of the player experience. Chatzitofis et al (2015) [11] implemented a home-based rehabilitation system for cardio vascular diseases using Kinect and body worn inertial sensors. The users need to start the game by warming up, and then they need to execute the assigned movement. Visual feedback is generated on the screen to help the user to optimize the movement. The system was evaluated with 6 patients. Finally, Lozano-Quilis et al (2014) [12] implemented an augmented reality system for multiple sclerosis using the Kinect. The system is called RemoviEM and includes 3 game exercises (TouchBall, TakeBall, and StepBall). In total, 11 patients tested the ability of the system to encourage players to perform exercises. The results were collected through a questionnaire and showed that patients accepted the system and felt safe and secure while playing.

Parkinson disease has been a subject of interest among serious game projects. Yu et al (2011) [13] developed a real-time Parkinson mediated rehabilitation environment. They implemented a system applied in a clinical space to treat Parkinson disease symptoms by improving the patient’s ability to reach and step as far and as fast as possible. Patients are required to execute repetitive and variable tasks in order to learn new movement patterns and to perform the transition from one movement to another by performing mixed and multiple tasks. A virtual avatar is shown on the screen that mimics the patient’s movements. However, the system was never tested on Parkinson disease patients. Assad et al (2011) also investigated the use of serious games for Parkinson disease patients, and they implemented a series of games that use the Sony PlayStation EyeToy as a motion capture tool [14]. Four different Parkinson disease adapted games were developed and tested by 13 Parkinson disease patients. The system was rated using a questionnaire completed by the patients after performing the exercises. This study concluded that the patients enjoyed the exercises. Paraskevopoulos et al (2014) developed serious games adapted to Parkinson disease patients [15]. They defined a guideline to successfully design serious games adapted to Parkinson disease through a detailed literature review of related works, and developed 2 games using the Wii Mote and the Kinect camera. They tested the games on 5 Parkinson disease patients and concluded that serious games have the potential to increase the level of engagement for such patients.

Another rehabilitation field that has been studied is stroke rehabilitation. Cho et al (2014) developed a proprioception rehabilitation system for stroke patients [16]. The user moves a connected cylinder to interact with the game. The objective was to hold the connected cylinder under a table to move the virtual cylinder from an initial position to a destination position. The study was tested with 10 healthy subjects and 10 stroke patients and showed significant improvement in patients. However, this improvement might have been attributed to patients becoming accustomed to the game. Another system used a commercial Wii Fit game and 2 Wii balance boards to adapt the games to stroke survivors [17]. Each balance board captures the center of pressure of the leg. The weak leg’s signal is multiplied by a higher weight than the healthy leg’s signal so that the patient applies more load on the weak leg. The system was tested on 3 stroke survivors (2 participants and 1 control) and showed that after 7 to 12 sessions, the patients began to rely more on their weak legs and began to tend to normal load ratios observed in healthy subjects. Ibarra Zannatha et al (2013) also developed a serious for game stroke rehabilitation using the Kinect camera, electromyography (EMG) sensors, and a humanoid robot [18]. The system consists of 4 games for the upper limbs. This system was not tested on stroke patients.

Literature showed that serious games have been intensively developed for general health and specific disease management. One of the most important aspects of serious games is the game playing scenario to motivate the patient. Moreover, user acceptability also plays an important role in promoting this new technology to clinical practice. Finally, the user security aspect needs particular attention to avoid new clinical complications for patients. Different game systems have been developed and tested. There is still lack of development and evaluation consensus guidelines to achieve these important aspects. It is important to note that the game performance depends on the designed scenario. Some authors have attempted to propose specific guidelines for game development-based learning [19] or for Parkinson disease rehabilitation [15]. However, methodologies and best practices related to the development of customized serious games for musculoskeletal disorders to recover complex joint and muscle functions are still lacking. Thus, the objective of this work was to develop specific game scenarios and to evaluate them with a panel of musculoskeletal patients, to propose game development and evaluation guidelines. In particular, specific games for the functional rehabilitation of musculoskeletal disorders were developed and evaluated using the proposed approach. Hence, the usefulness of the developed games was quantified. Discussion on the usefulness of the proposed guidelines according to the literature was also provided.

## Methods

### Development of Serious Games

**Figure 1:**
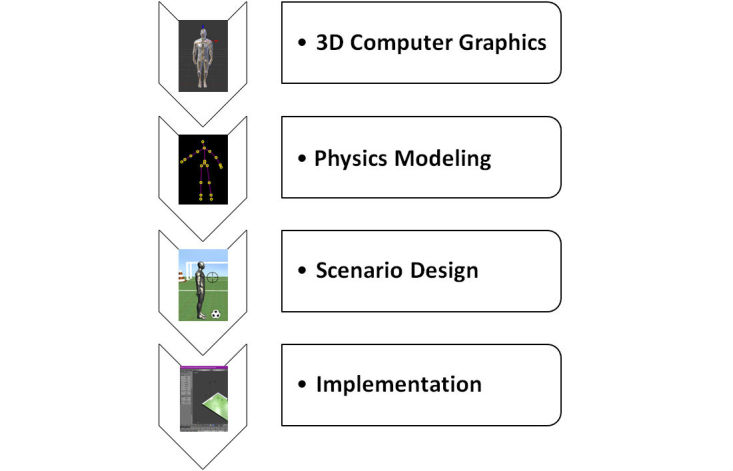
Rehabilitation-oriented serious game: development guideline.

**Figure 2:**
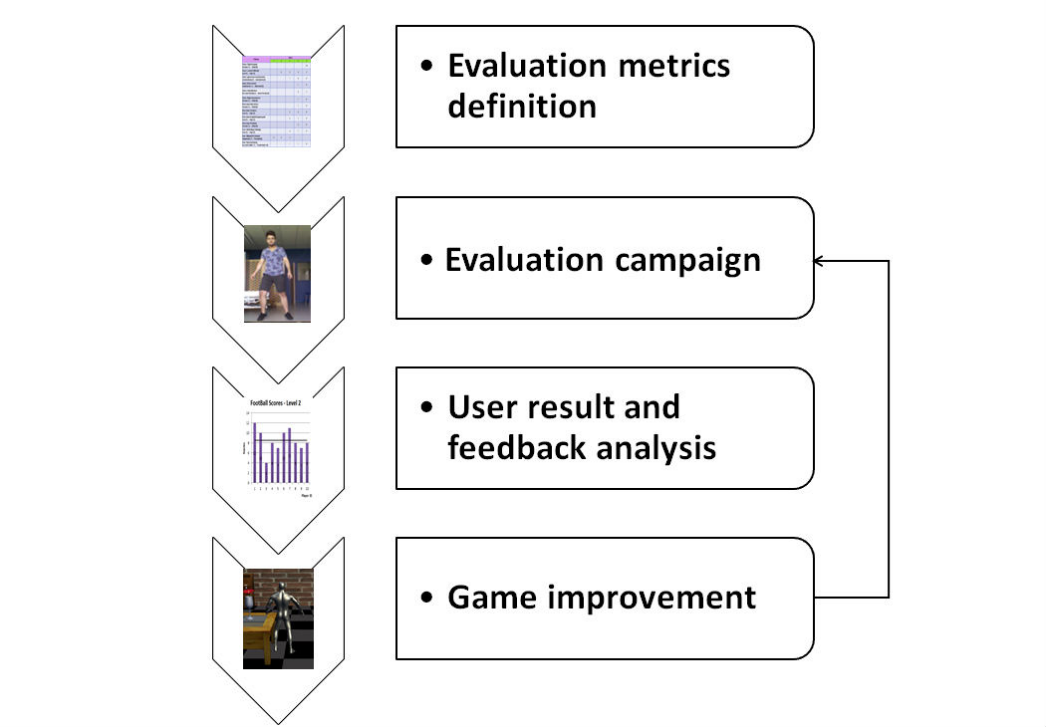
Rehabilitation-oriented serious game: evaluation guideline.

The development of serious games for functional rehabilitation of musculoskeletal disorders is a complex engineering task. To deal with such complexity, a two-stage workflow was proposed. The first workflow relates to the development guideline (Figure 1), whereas the second workflow concerns the evaluation guideline (Figure 2). The development workflow includes the selection of three-dimensional (3D) computer graphics technologies and tools, the modeling of physical aspects, the design of rehabilitation scenarios, and the implementation of the proposed scenario. This workflow aims to design fun but useful game scenarios to motivate end users to perform functional rehabilitation tasks. The evaluation guideline consists of the definition of the evaluation metrics, the execution of the evaluation campaign, the analysis of user results and feedbacks, and the improvement of the designed game. Finally, the improved game is reevaluated in a closed-loop technique. This user-centered game design approach allows different users (eg, patients and medical experts) to participate actively in the design and evaluation stages. Note that the second workflow focuses on the evaluation of user acceptability and security aspects when using this new technology. The development of these guidelines was performed using determinant framework [20]. This theoretical approach has been commonly used to determine what important factors influence implementation outcomes. Thus, eight determinants (3D computer graphics technologies, physics modeling, scenario design, implementation, evaluation metrics definition, evaluation campaign, user result and feedback analysis, and game improvement) were hypothesized to influence the implementation outcomes of the development and evaluation of rehabilitation-oriented serious games. In fact, these components aim to cover necessary methodologies and best practices for developing customized serious games for musculoskeletal disorders and evaluating them. The choice of these components is based on our experiences gained from literature analysis and also from our preliminary studies on functional rehabilitation using serious game technologies [7,8].

### Case Study for Musculoskeletal Disorders

#### Rehabilitation-Oriented Serious Game: Development Guideline

This subsection describes the work done using a proposed development guideline for creating specific serious games for functional rehabilitation of musculoskeletal disorders.

##### Three-Dimensional (3D) Computer Graphics Technologies and Tools

The selection of available computer graphics technologies and tools plays a crucial role in the success of the rehabilitation game. To ensure a user-friendly, human-system interaction, cutting-edge technologies benefiting the most recent progress of ICT solutions need to be used. In this study, open source Blender design software (Neo Geo) was selected for human body modeling. XNA Game Studio (Microsoft) was selected as game engine. Microsoft Kinect camera was selected as human motion capture tool. Computer screen was used as human-system interface. The pertinence of these technological choices has been proven in our previous studies [7-8,21].

##### Physics Modeling

A 3D avatar model was developed using Blender design software to represent the human body. This is a 3D surface mesh model including a collection of vertices, edges, and faces that defines the external shape of the human body. Moreover, an internal skeleton structure was also created to define body segments (eg, thigh and leg) and their interaction (eg, joint) during the motion [21]. Game environments and interaction objects were also designed and implemented using Blender design software.

**Figure 3:**
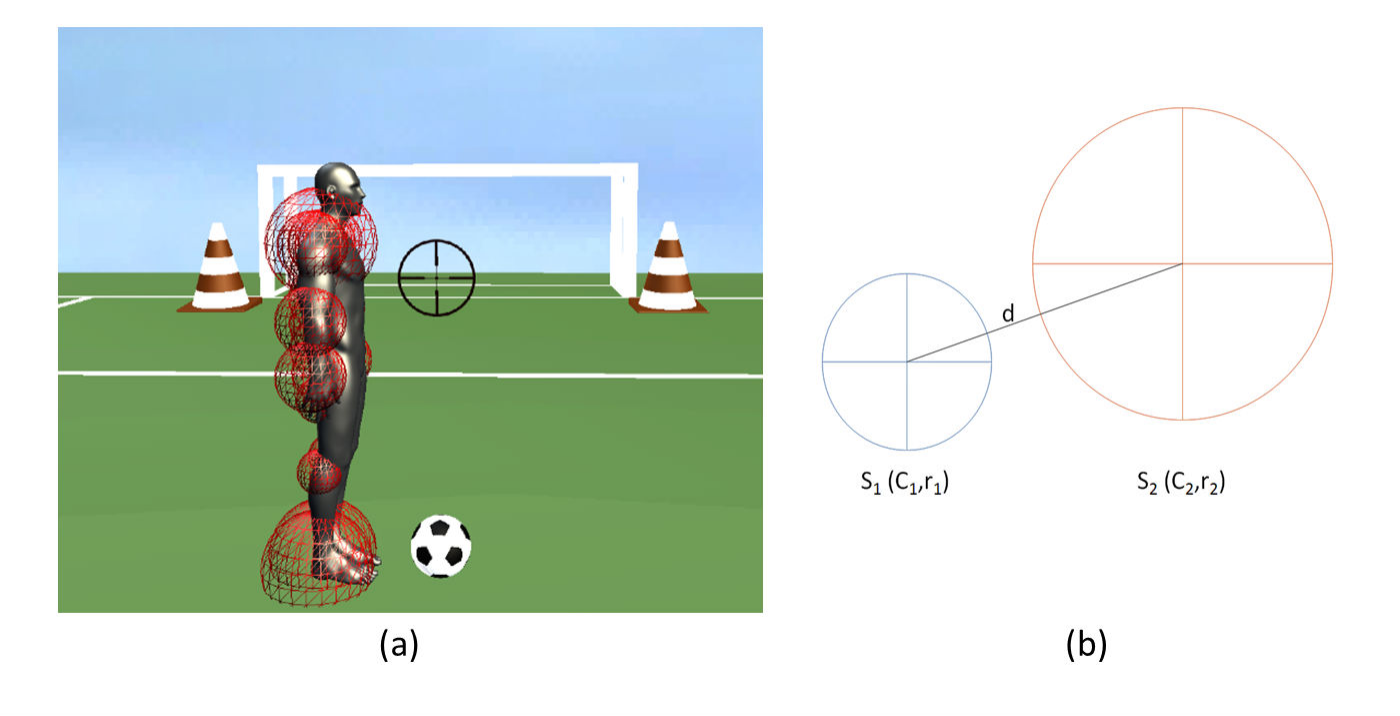
Illustrations of the association of collision spheres to avatar bones (a) and object collision detection principle (b).

Games with 3D interactive objects need to establish interaction rules between them. An algorithm was designed and implemented to detect collisions between objects within the scene. The challenge was to find a way to differentiate between the detection between different avatar bones and 3D objects; therefore, we created spheres around each bone of the body (Figure 3. Note that the radius and positions of these spheres are adjustable to a specific subject body. The assessment of the collisions is done by calculating the distance between the spheres of objects and bones (Figure 3).

Let S_1_ be a sphere with a 3D center C_1_(C_1x_, C_1y_, C_1z_) and a radius r_1_, and S_2_ another sphere with center C_2_(C_2x_, C_2y_, C_2z_) and radius r_2_. The distance between the 2 centers of the 2 spheres is d drawn in Figure 3 and is computed using the following equation:

d=[(C
_1x_-C
_2x_)
^2^+(C
_1y_-C
_2y_)
^2^+(C
_1z_-C
_2z_)
^2^]
^1/2^

This distance is computed between every 2 objects at each updated iteration during the game. If d is found to be less than the sum of the 2 radiuses r_1_ and r_2_, a collision is detected, and the game reacts to it by a certain preprogramed reaction.

##### Scenario Design

Two task-oriented game scenarios (football and object manipulation) were designed and implemented. The football game aims at practicing body orientation and lower limb motions, allowing the rehabilitation of spinal and lower limb systems. The object manipulation aims to practice the upper limb and lower limb motions with a focus on the detailed hand skill. The description of each game is given in the following paragraphs.

###### Football Game

**Figure 4:**
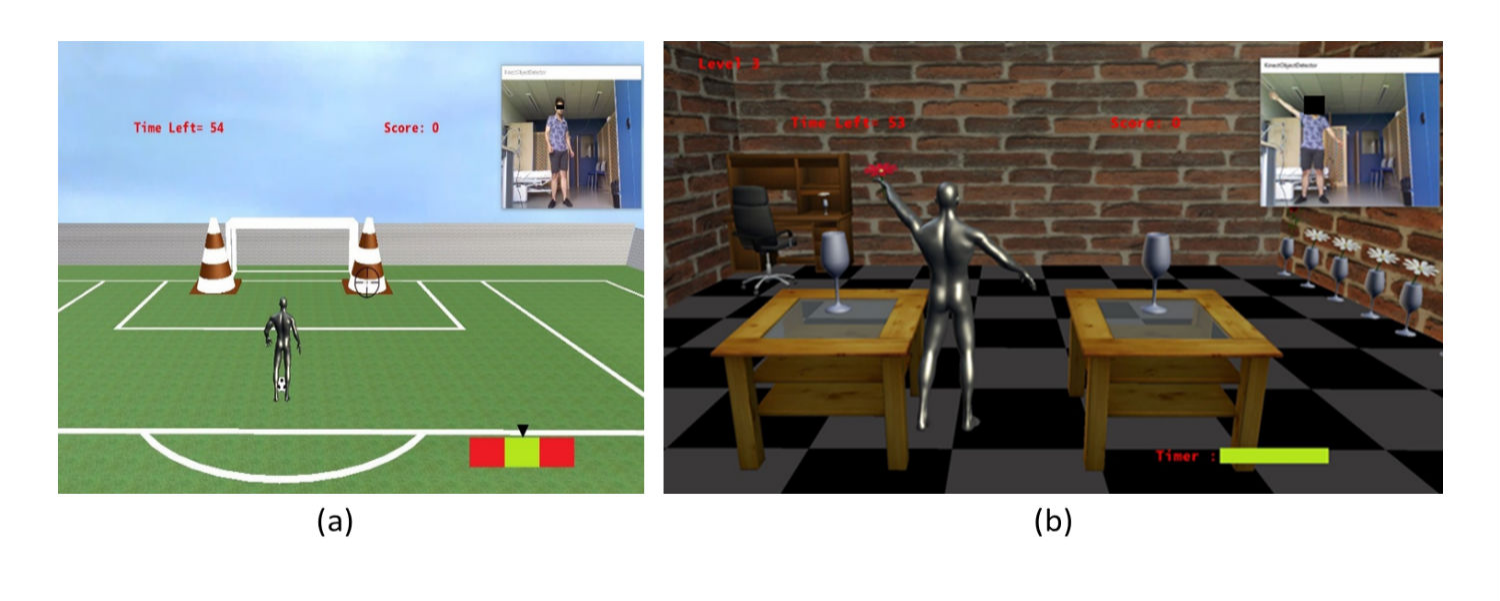
Football game (easy level) (a) and object manipulation game (hard level) (b).

This game requires the player to execute many consecutive gestures. First, players have to stand in front of the Kinect and the computer screen. Then, they need to target the left or right cones by pivoting their body (Figure 4). Once the target is reached, the player has to verify that the pointer in the bottom right corner of the screen is in the green zone. If the pointer is green, they kick the ball to hit the cone and score one point. Otherwise, if they kick while the pointer is red, the ball will miss. When the cone is hit, the user needs to pivot back to the original position to get another ball. A point is awarded for every cone hit. We developed three levels of difficulty because patients playing the game might be in different phases of their rehabilitation. Using the different developed levels, experts can configure the difficulty of the exercises to be executed by their patients according to the rehabilitation progress. In the easy level, the cones are big and the green or red pointer is slow. The medium level decreases the size of the cones. Finally, to make it harder, the pointer will move faster on the hard level. Experts can also define the duration for each exercise, which gives them more control over the rehabilitation program. This game aims at the rehabilitation of several parts of the body. It targets balance, since the users rotate to target a cone. In addition, it includes a decision-making action, since players have to verify the pointer position. Finally, the lower limbs are also affected, since the patient has to kick the ball. It is noted that a soccer stadium was designed for this specific rehabilitation game.

###### Object Manipulation Game

In this scene, the user needs to take a flower from the given vase and put it in the other one (Figure 4). They repeat the same actions from right to left until the game-time expires. Three levels of difficulty (easy, medium, and hard) are defined. In the first level, the virtual avatar is fixed between 2 tables and can only move their hands. In particular, the player is rewarded 4 points for a combination of 3 successive gestures: take the flower with the first hand from the first vase, switch the flower to the second hand, and put the flower in the second vase. The second level of this game requires the player to move left and right while a certain distance separates the tables. Therefore, players have to move one step left and then get the flower. They switch it to the other hand and then move one step to the right in order to put the flower in the other vase. Finally, the third level of difficulty is similar to the second one but the challenge is to put the flower in the other vase before the expiration of a timer that appears on the bottom of the screen. This game targets several parts of the body. The upper limbs are targeted in all the levels, whereas the lower limbs are targeted only by the second and third levels. Moreover, the third level targets lower limb movement speed recovery, since the timer would force the users to move quicker. It is noted that a surrounding living room was designed for this specific rehabilitation game.

##### Implementation

Visual Studio.Net, with C# programming language, was adopted for image acquisition and processing, body tracking, object manipulation, as well as for the development of graphical user interfaces (GUIs).

#### Rehabilitation-Oriented Serious Game: Evaluation Guideline

This part presents the work done, as well as outcomes issued from the application of the proposed evaluation guideline for assessing the developed games.

##### Definition of Evaluation Metrics

The game-playing performance was evaluated by the points acquired at the end of each scenario. For the usage acceptability aspect of the designed games, a questionnaire was defined. At the end of each game scene, players were required to fill out a questionnaire. The questionnaire consists of 13 questions for each specific game scenario. The feedback focuses on the game, exercise, and user aspect. For the game, the objective, the level of difficulty, the ignorance of achievement, the attractiveness of the 3D environment and GUI, and the game management (begin, end) were investigates. For the exercise, the game instructions, the variation of scenarios, the suitability of the game to the goal, and the clearness of the feedback were examined. For the user, the motivating challenge, the possibility to make mistakes, and the security feeling were investigated.

##### Evaluation Campaign

The developed game scenarios were evaluated by a normal healthy group (10 subjects: 6 males and 4 females with a mean age of 26.8 [standard deviation, SD 5.65]), to ensure the security condition, and then evaluated by a population of 20 pathological subjects (13 males and 7 females with a mean age of 49.75 [SD 18.68]) at the “Centre Hospitalier Universitaire de Limoges” (France). The patient group included different musculoskeletal disorders (3 amputee patients, 8 hemiplegia patients, 1 hereditary spastic paraplegia patient, 1 patient with ankle arthrodesis, 1 stroke patient, 1 patient with shoulder capsulitis, 1 patient with low back pain, 1 patient with carpal tunnel, 1 patient with prosthesis, 1 patient with muscle disease, and 1 patient with walking difficulty due to a car accident). Each participant signed an informed consent agreement before playing the rehabilitation games. It is important to note that the execution of rehabilitation serious game was monitored by clinicians, to ensure the ability and the security of the patients when using this new rehabilitation tool. Each healthy subject was asked to play every level of difficulty of each game, which means a total of 6 trials per subject. Some patients were not able to try all levels or even one of the two games due to the severity of their state (amputation, leg prosthesis, and paralyses). Medical experts were given the decision to accept or decline the participation of their patient in a game or a level of a certain game. Therapists accompanied their patients by standing behind them and supporting them, to ensure their security. The duration of each game level was around 60 seconds. A rest time of around 2 min was also allowed for each participant when necessary (ie, recovery from fatigue) after each game execution. The total time of the test for one subject was equal to 20 min approximately.

## Results

### User Result and Feedback Analysis

**Figure 5:**
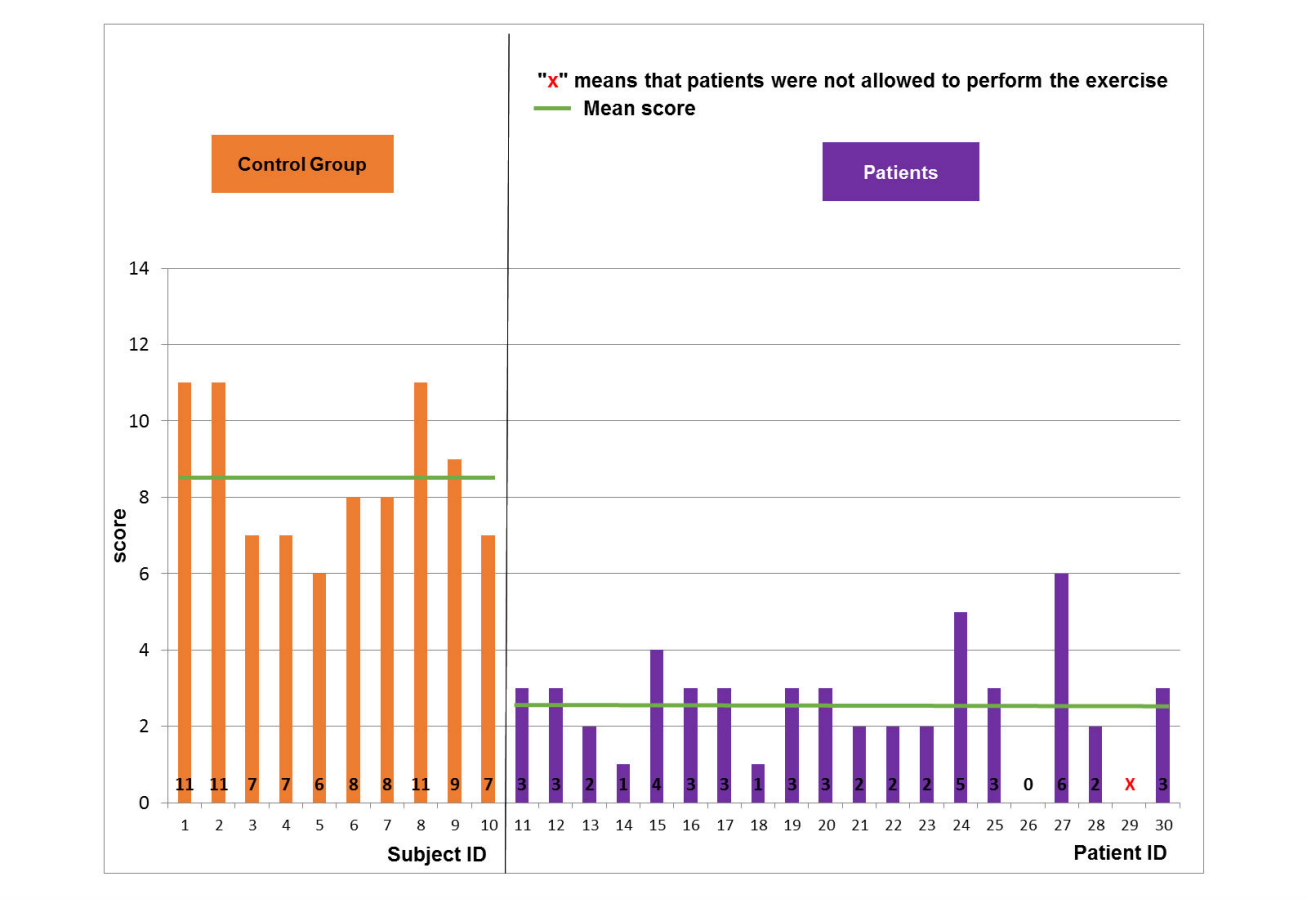
Game performance: patient group vs. healthy control group: easy level of the football scenario.

**Figure 6:**
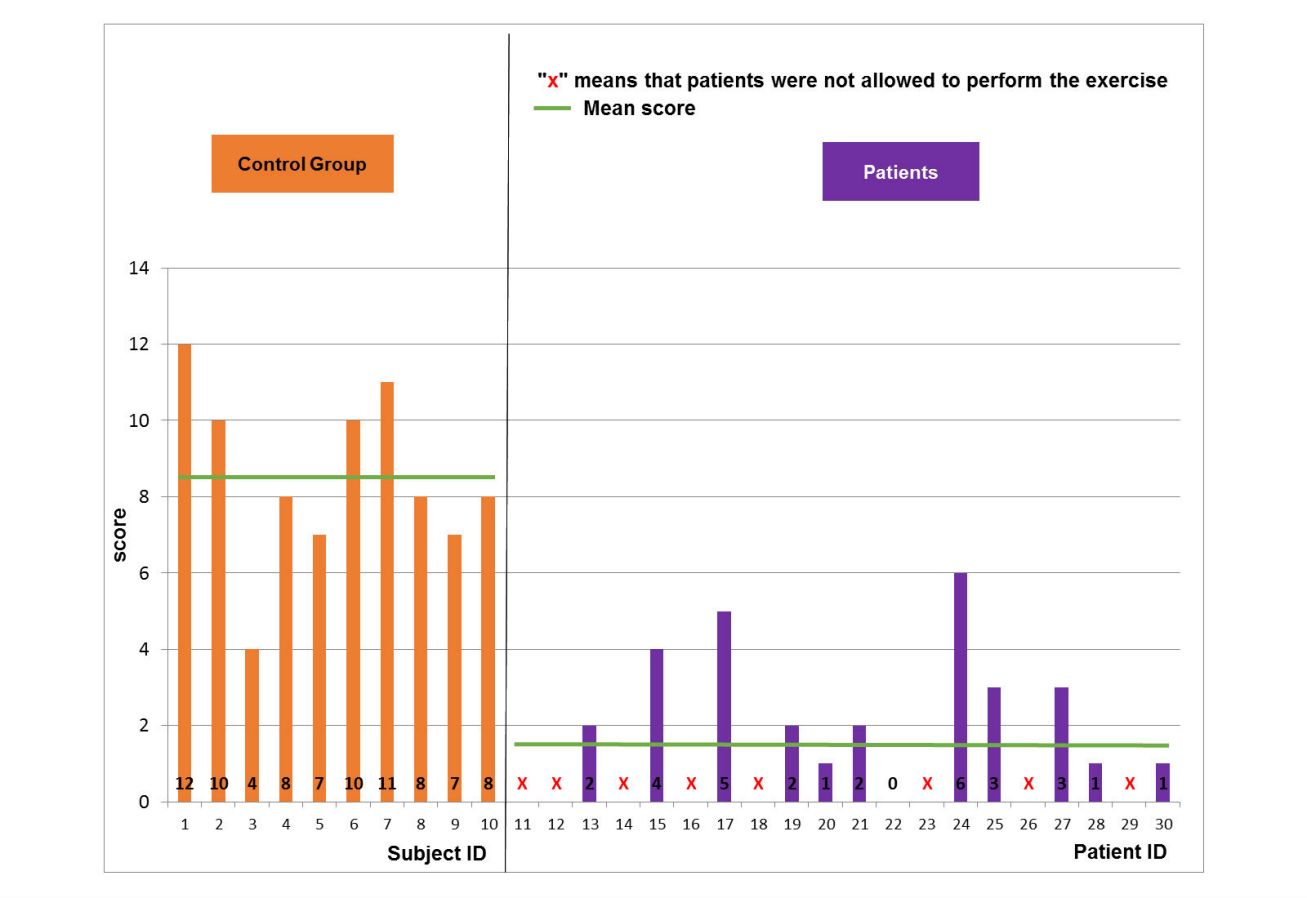
Game performance: patient group vs. healthy control group: medium level of the football scenario.

**Figure 7:**
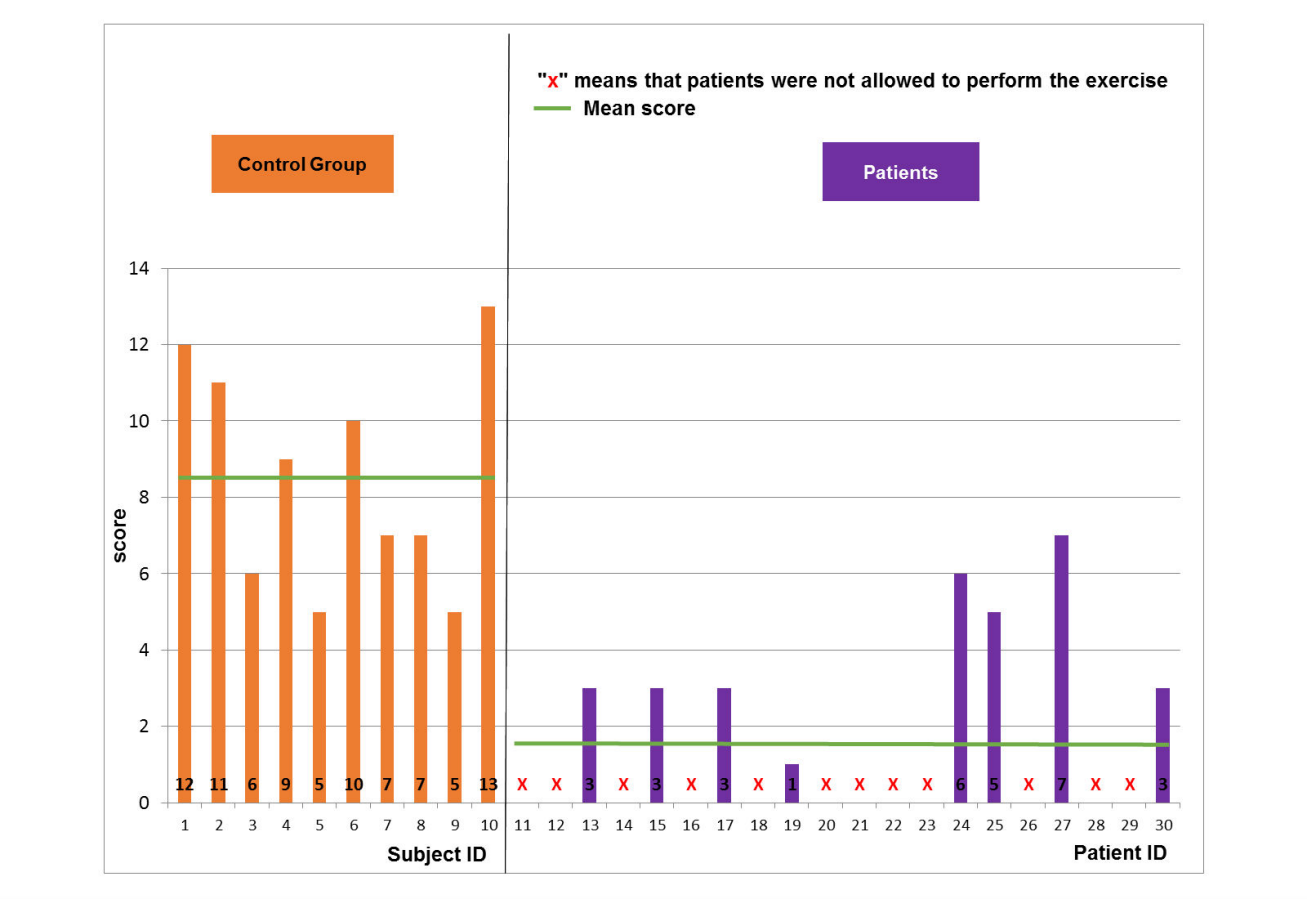
Game performance: patient group vs. healthy control group: hard level of the football scenario.

For the control group, the scores did not change so much when increasing the level of difficulty for the football scenario (Figures 5-7). The mean and SD scores of the easy, medium, and hard levels were 8.5 (SD 1.8), 8.5 (SD 2.2), and 8.5 (SD 2.7), respectively. Maximal scores were 11, 12, and 13 for the easy, medium, and hard levels of difficulty, respectively. Note that when a score is achieved, this means that the player finished a game with all requirements. Statistical test (*t*-test, implemented in Matlab R2010b software [The MathWorks Inc.]) showed no significant difference. In particular, some subjects (ID4 or ID6) increased their score when enhancing the level of difficulty. According to the healthy control group, the performance of the pathological population was significantly (*t*-test, *P*<.005) lower for all levels of difficulty (Figures 5-7). The mean and SD scores of the easy, medium, and hard levels were 2.7 (SD 1.3), 2.5 (SD 1.7), and 3.9 (SD 1.8), respectively. Maximal scores were 6, 6, and 7 for the easy, medium, and hard levels of difficulty, respectively. In particular, some patients (ID17 or ID24) increased their score when enhancing the level of difficulty. However, the number of the patients able to perform on harder levels was reduced from 19 patients for easy level to 8 patients for the hard level.

**Figure 8:**
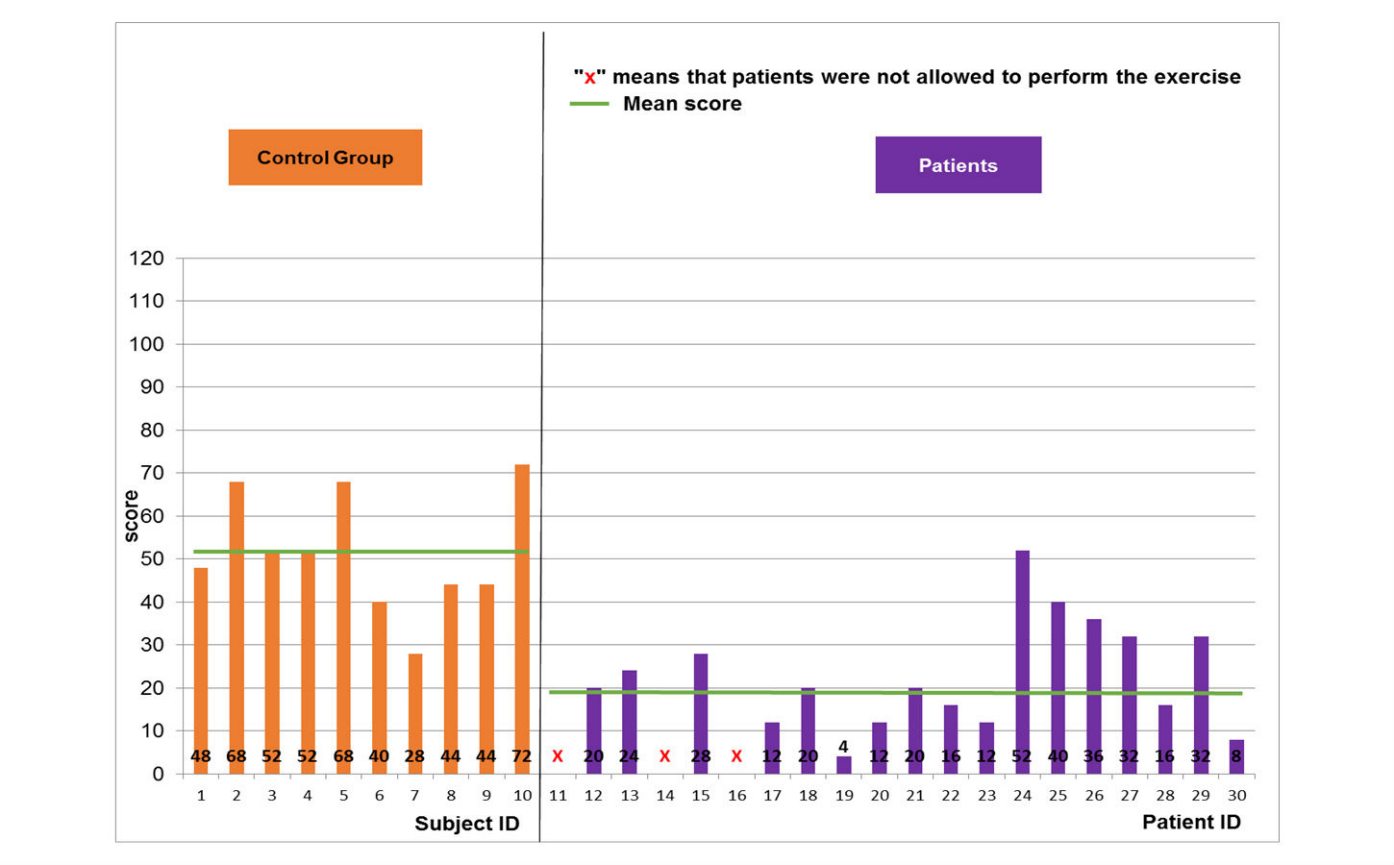
Game performance: patient group vs. healthy control group: easy level of the object manipulation scenario.

**Figure 9:**
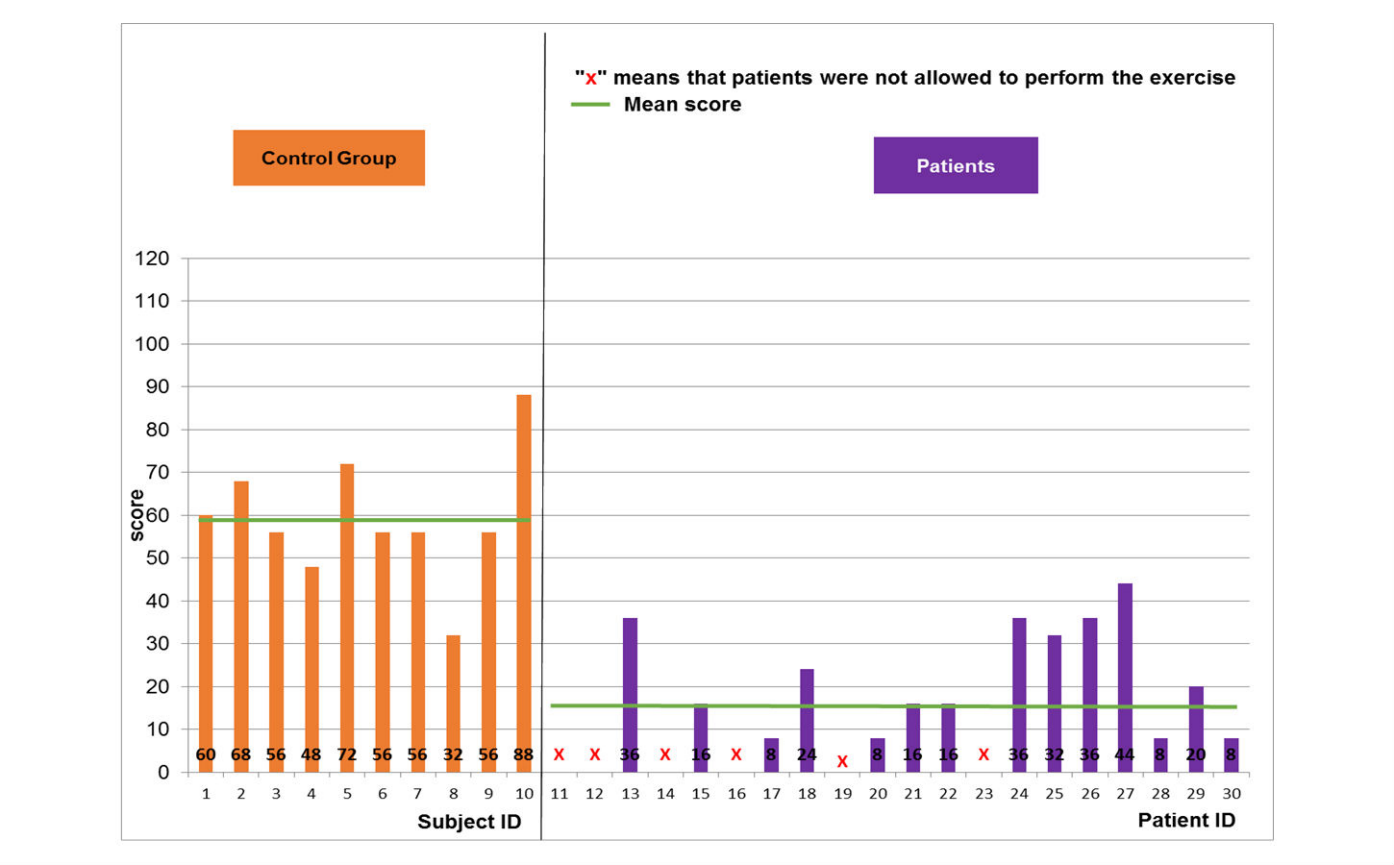
Game performance: patient group vs. healthy control group: medium level of the object manipulation scenario.

**Figure 10:**
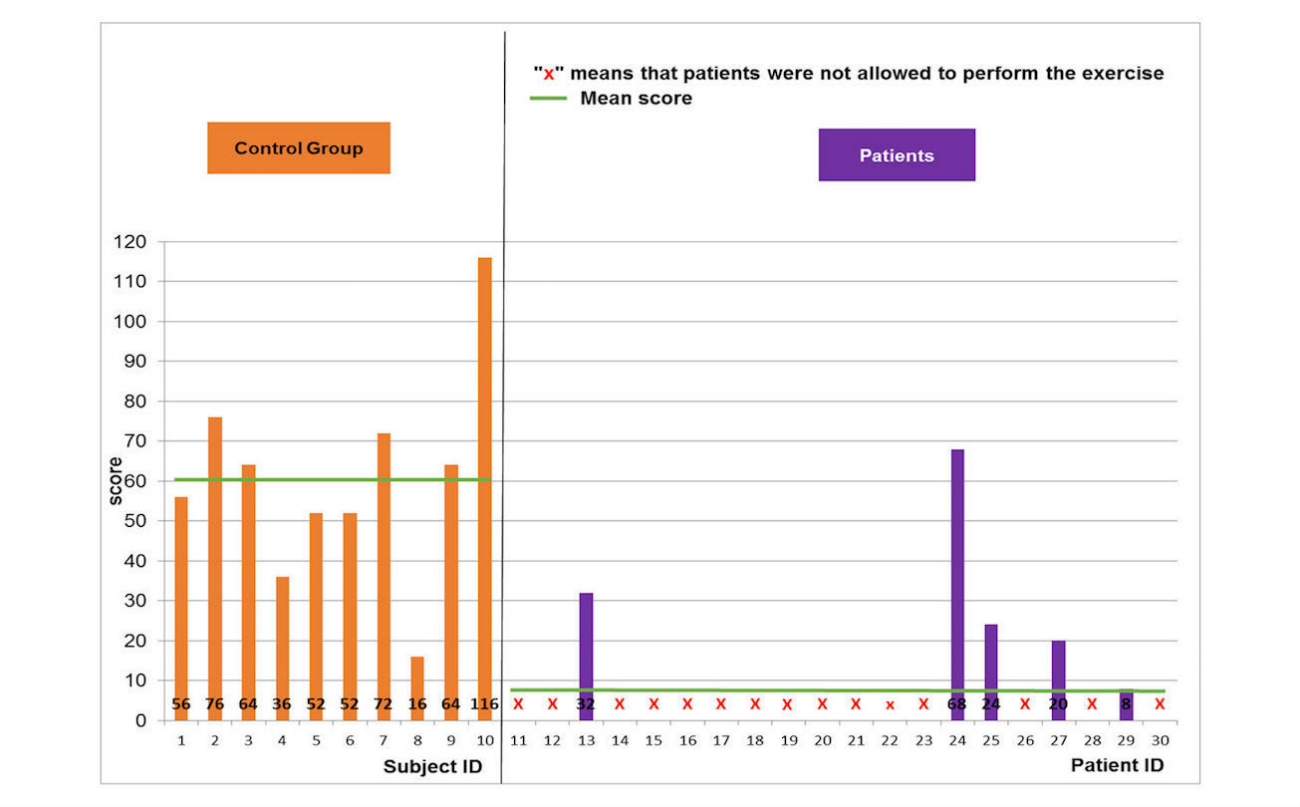
Game performance: patient group vs. healthy control group: hard level of the object manipulation scenario.

Regarding the object manipulation game, the same results were noted (Figures 8-10). The normal population showed mean and SD scores of 51.6 (SD 13.3), 59.2 (SD 14), and 60.4 (SD 25) for the easy, medium, and hard levels, respectively. Maximal scores were 72, 88, and 116 for the easy, medium, and hard levels of difficulty, respectively. The pathological population showed mean and SD scores of 22.8 (SD 12.3), 22 (SD 12.2), and 25.3 (SD 21.7) for the easy, medium, and hard levels, respectively. Maximal scores were 52, 44, and 68 for the easy, medium, and hard levels of difficulty, respectively. Thus, the performance of the pathological population was significantly (*t*-test, *P*<.05) lower than that of the normal population. The number of the patients able to perform harder levels was also reduced from 17 patients for the easy level to 5 patients for the hard level.

For the responses to the questionnaires, 29 users (patients and healthy subjects) rated the football game, and 27 rated the object manipulation game.

Regarding the user acceptability of the evaluated games, all healthy subjects found the 2 developed games motivational, attractive, and challenging. A synthesis of the patients’ responses to the football game questionnaire is depicted in Table 1. Moreover, they enjoyed all the levels of difficulty. Note that the answers about the accuracy of the human movements’ detection varied. That can be interpreted by the limitations of the Kinect due to occlusion of limbs, which could affect the accuracy of movement detection. Most of the participants assumed that they were comfortable with the system, whereas some patients, having balance disorders, worried about some levels of difficulty. Finally, there were no risks and accidents associated with the execution of these 2 games not only for the normal population but also for the pathological population.

### Game Improvement

Finally, players were asked to give some specific comments on this project and the developed games. Comments and suggestions from the patient groups are summarized as follows:

Interesting game and this game needs to be developed in bigger scales.

The games are amusing, motivational and not bad at all. It made me really move my legs.

The football scene is excellent. I am a football fan and I watch all the games.

I recommend you to force the player to hit the left cone at first and then rotate towards the right cone. This improves the efficacy of spine rehabilitation.

In my opinion this can really help patients. Even if I am not a florist!

The exercises are adapted to rehabilitation at the final stages.

The project is suitable for younger players.

The project is very fun, helps in performing rehabilitation while enjoying it. It should please young and old people.

Very attractive games.

Very interesting project for movement coordination.

The avatar's movements should be improved.

Difficult but interesting. More games need to be developed.

Based on these suggestions, our game scenarios were updated to take them into consideration. Note that only technical improvement feedbacks were considered in the updated version. In particular, the order of the football game, as suggested in the third comment above, was redefined to adapt to the rehabilitation of spinal patients. Moreover, avatar’s movement has been improved by using multi-sensor fusion approach [22]. Some patients did not try the football game because they could not stand up on their feet. This could be an initiative to create exercises for patients sitting on wheelchairs in the future version of our serious game system.

**Table 1:** Patients’ responses to the football game questionnaire.

Criteria	Rank				
	1	2	3	4	5
Game: Objective/goal	1		1	2	15
Unclear (1) → Clear (5)					
Game: Level of difficulty	3	4	7	2	3
Low (1) → High (5)					
Game: Ignorance of achievement			3	2	14
Unawareness (1) → Awareness (5)					
Game: Environment	1		1	5	12
Unattractive (1) → Attractive (5)					
Game: User Interface			3	2	14
Not user-friendly (1) → User-friendly (5)					
Game: Beginning and end	1		1	3	14
Unclear (1) → Clear (5)					
Exercises: Instructions	1			1	17
Unclear (1) → Clear (5)					
Exercises: Variation		3	1	6	9
Low (1) → High (5)					
Exercises: Suitable for game goal		2	5	3	9
Low (1) → High (5)					
Exercises: Feedback		1	3	4	11
Unclear (1) → Clear (5)					
User: Motivating challenge	2	2	1	4	10
Low (1) → High (5)					
User: Mistake permission	6	2	7	1	3
Impossible (1) → Possible (5)					
User: Security feeling			3		16
Uncomfortable (1) → Comfortable (5)					
Total	15	14	36	32	144

## Discussion

### Principal Findings

Serious gaming technologies target audience ranging from young to adults to the elderly population. The objective of this work was to propose development and evaluation guidelines of serious games for musculoskeletal disorders. The simplicity and challenging aspect are the main advantages of this new technology. Research studies have proposed some interesting solutions over the past decade for the “gamification” approach [23-26]. Seaborn and Fels (2015) reviewed and defined “gamification” as the use of game elements to execute nongame tasks in a game-like environment [27]. In particular, Wattanasoontorn et al (2013) conducted a survey on serious games for health, and they showed that general health was the most targeted by serious games, whereas stroke disease comes in second place [28]. Furthermore, the mouse was the most used interaction tool, mostly used for cognitive rehabilitation. Kinect and Wii Mote cameras tied in first place among motion capture tools. Serious game for functional rehabilitation has become a potential solution to improve the traditional rehabilitation practice [29-32]. Generally speaking, user acceptability was high for some developed games [31]. Clinical improvements over time were also noted [30-32]. However, this new rehabilitation scheme needs to be used with caution because of some negative results. For example, Bower et al (2015) reported minor increases in pain for some participants. Particular attention was also noted for cognitive function and motor impairment patients when using virtual reality rehabilitation games [31]. Thus, the development of rehabilitation games should be done in a well-controlled manner. In previous works, there is no available development guideline for this new rehabilitation scheme. In this study, we proposed a specific task-oriented development guideline to create attractive, motivational, and safe rehabilitation games. The experience that we got from the case study of musculoskeletal disorders showed the usefulness and applicability of the established task-oriented development guideline. Furthermore, an evaluation guideline was also established to propose the common steps toward an objective and quantitative evaluation process. The case study showed the applicability and usefulness of the proposed guidelines in real condition. It is expected that this study may contribute in the definition of customized guidelines for developing and evaluating serious games for musculoskeletal disorders.

Regarding our case study, patients’ scores were lower than those of the healthy group. Some of them were not able to play the football scene because of their amputation. Others could not try the object manipulation scene because they cannot move their hands at all. In general, all of them accepted the challenge and wanted to participate in this study. Hemiplegic patients were the top testers among all patients. Medical doctors and physiotherapists thought that these task-oriented games were more adapted to this particular disorder. Previous study came to the same conclusion about the use of the task-oriented games for these patients [16,17]. We can see that all hemiplegic patients were able to try at least one level of difficulty from each game, and their achieved scores depended largely on the severity of their disorder. Amputees tried our system and showed great motivation even though they failed to achieve high scores; they felt the challenge even in the absence of any achievement. Moreover, the Kinect had some difficulty recognizing the shape of their body, which might influence the virtual avatar’s behavior. Overall, patients’ results depend on the state of each patient. Moreover, even though the difficulty of the games increased, some patients and healthy subjects achieved higher scores even at the hard level of difficulty. This might be explained by the fact that the designed games stimulated the user motivation. Thus, they felt the challenge to perform better when they got familiar with the game. However, more quantitative measurements on user’s motivation need to be performed to confirm this finding. Moreover, the increasing scores occurred for the favorite game for each patient. Thus, the choice of the game scenario for the profile of each patient may potentially enhance the achieved scores.

The design of rehabilitation game scenarios plays a crucial role in the success of the serious game for health. The game scenario must not only be attractive but also needs to be clinically useful. In this study, a task-oriented rehabilitation game scenario was proposed. The football and object manipulation games respond to the challenging objective: patient practices rehabilitation exercises without recognizing that it is a rehabilitation exercise when playing the game. Thus, the football game allows the player to practice the two motor tasks (body rotation motion and the leg motion) and two decision-making actions (observation of time and identification of right moment). The object manipulation game allows the player to practice two motor tasks (leg and arm motion) and two decision-making actions (localization of rose or vase, and observation of time). This study suggests that rehabilitation game scenarios should be designed, implemented, and evaluated with similar strategies. The outcome of the case study confirmed the robustness and effectiveness of this strategy.

The design of a motivating, challenging, and safe serious games for functional rehabilitation requires particular attention on the development and evaluation processes. This study proposes useful guidelines to achieve this objective. Thus, the development and evaluation of the 2 developed games (football and object manipulation) followed the proposed guidelines. In general, a guideline is defined as a principle to determine a set of actions in a standard way. This study aimed to propose a coherent set of development and evaluation steps for rehabilitation-oriented serious games for musculoskeletal disorders. It is expected that this proposition may help define a development and evaluation consensus in the health-oriented serious games community. It is important to note that some published works already followed these guidelines [9,10,12] but other works did not conduct some important steps like the improvement of game from user feedback [16] or the evaluation on patients [18]. Thus, this study may serve to highlight the important steps to develop and evaluate a serious game for musculoskeletal disorders.

Our developed system used the Kinect camera as motion capture sensor. Currently, the virtual avatar imitates player movements correctly. However, clinical experts require more precision in analyzing the joint behavior during the exercise. It is well known that the accuracy of this device is limited for joint angle estimation. A deviation range of 11° to 14° was noted for the knee joint motion [8,33]. To overcome this drawback, a fusion process between the Kinect and inertial sensors, placed on the part of the body where experts require more precision, was investigated in another work to achieve a better estimation of joint angles [22]. However, the use of only Kinect camera leads to the feasible and potential translation of such a rehabilitation game into clinical routine practice, especially in a home-based setting thanks to the low cost and portable nature of this specific device. More complex sensors need to be optimized before they are used in a clinical setting. In particular, within the context of a “game,” the precision may be sacrificed for the portability and ease-of-use criteria.

### Limitations

The main limitation of the 2 developed games is the lack of evident cognitive actions, which could maximize the effect of game outcomes to better manage the functional rehabilitation of musculoskeletal disorders with cognitive impairment [34,35]. Thus, a new rehabilitation game will be investigated to integrate clear cognitive aspects into the game scenario. Thus, cognitive actions may help detect visuo-spatial memory and propose an appropriate rehabilitation program [36]. Moreover, the evaluation of the effectiveness of these serious games will be performed during a long-term campaign to confirm their clinical relevance. Finally, in this study, the user questionnaire was based on the one defined previously [8]. This questionnaire covers many aspects including the game, the exercise, and the user. However, the user engagement aspect is still simple in the used questionnaire. Thus, the use of a validated questionnaire that focuses more on the user aspects to analyze the game engagement will be performed in the future [37].

In summary, this study is an explanatory work aiming to show the usefulness and applicability of the proposed guidelines and associated serious games for functional rehabilitation of musculoskeletal disorders. However, more investigations such as a long-term evaluation campaign for effectiveness analysis and a more quantitative analysis on the user‘s engagement in the games are needed to fully validate these guidelines [38].

### Conclusions

Development and evaluation guidelines dedicated to serious games for health were established in this study. The case study showed the effectiveness and usefulness of these guidelines and associated games. The developed serious game system used the Kinect camera to allow users to interact with two 3D environment scenes (football and object manipulation). Healthy subjects and patients enjoyed the games and found them challenging and amusing. In this work, we concentrated on the assessment data of the developed games. In perspective, the effectiveness and clinical relevance of these games will be studied through a long-term evaluation campaign. And, in the case of positive outcomes, this new rehabilitation game may be translated into clinical routine practice in the near future for the benefit of patients with musculoskeletal disorders.

## Abbreviations

3D: three-dimensional
EMG: electromyography
GUI: graphical user interface
ICT: information and communication technology
SD: standard deviation
